# Relationship between Physical Activity Levels and Academic Performance in Adolescents from Serbia

**DOI:** 10.3390/children11101198

**Published:** 2024-09-29

**Authors:** Tamara Ilić, Stefan Stojanović, Doroteja Rančić, Bojan Milenko Jorgić, Rareș Stănescu Cristian, Daniel Andrei Iordan, Codreanu Corneliu Mircea, Stoica Leonard, Ilie Onu

**Affiliations:** 1 Faculty of Sport and Physical Education, University of Niš, 18106 Niš, Serbia; tamarailic.fsfv@gmail.com (T.I.); pallantides64@gmail.com (S.S.); doroteja.r@yahoo.com (D.R.); bojan.jorgic@fsfv.ni.ac.rs (B.M.J.); 2 Faculty of Physical Education and Sport, National University of Physical Education and Sports of Bucharest, 060057 Bucharest, Romania; dcre@unefs.ro; 3 Research Centre for Physical Therapy and Rehabilitation, “Dunărea de Jos” University of Galati, 800008 Galati, Romania; corneliu.codreanu@ugal.ro (C.C.M.); leonard.stoica@ugal.ro (S.L.); 4 Department of Individual Sports and Kinetotherapy, Faculty of Physical Education and Sport, “Dunarea de Jos” University of Galati, 800008 Galati, Romania; ilie.onu@umfiasi.ro; 5 Department of Biomedical Sciences, Faculty of Medical Bioengineering, University of Medicine and Pharmacy “Grigore T. Popa” Iasi, 700454 Iasi, Romania

**Keywords:** exercise, scholarly achievement, high school students, sedentary behavior

## Abstract

Background/Objectives: Today’s high school students often engage in sedentary behavior by choosing passive activities during leisure time. Increasing research shows that regular physical activity (PA) provides benefits beyond physical health, especially important during childhood and adolescence. Our study investigated the association between physical activity levels and academic performance (AP) in Serbian adolescents. Methods: We selected a sample of 836 fourth-grade high school students (avg. 17.9 ± 0.7 years old) who completed the IPAQ (short-version) and the Academic Performance Scale. Data analysis was conducted using IBM SPSS 21.0. Due to non-normal data distribution, Spearman’s correlation was used and hierarchical regression analysis to assess the association between PA and AP. Results: The findings revealed significant correlations between vigorous, moderate and light PA, as well as overall units of metabolic equivalent of task (METs), with AP (*p*-values: 0.000, 0.005, 0.021, 0.004, respectively), although with weak correlation coefficients (0.127, 0.098, 0.080, 0.181, respectively). Vigorous PA influenced AP by 10.1%, moderate PA by 8.2%, and light PA by 11.4%. Conclusions: These results suggest that adolescents engaging in higher PA levels tend to have better AP. This finding indicates a substantial association between PA levels and AP, with both moderate and vigorous PA showing a positive correlation and influence. Further investigation is necessary to comprehensively grasp the link between light PA and AP.

## 1. Introduction

An increasing amount of research indicates that engaging in regular physical activity (PA) has benefits that go beyond an individual’s physical health [1]. These benefits are especially relevant throughout childhood and adolescence [2], as these are the life phases when healthy habits should be developed for prevention against diseases like obesity, diabetes and high blood pressure [3]. Adolescents today are part of the sedentarism problem because they prefer to spend their free time engaging in daily activities that require them to be passive for extended periods of time, like playing video games or spending hours in front of the TV, using computers or using cell phones [4]. In the past, high school students used to spend their leisure time engaging in more physically demanding games and sports, either recreationally or competitively [4]. But because of many factors, the habit has progressively faded to the point where there is less time that can be used for engaging in physical exercise and sports [4]. Despite the considerable health advantages associated with PA, the overwhelming majority of adolescents globally lead sedentary lifestyles [5]. Global statistics from 2016. suggest that roughly 77.6% of boys and 84.7% of girls between the ages of 11 and 17 do not engage in sufficient PA [5], with evidence suggesting a decline in PA levels throughout adolescence [6]. Moreover, there has been a trend of decline in agility [7], explosive strength [8], speed [9] and cardiorespiratory endurance [10] among adolescents in the last two decades.

In addition, research indicates a connection between PA and children’s and adolescents’ AP in school [11,12,13]. This relationship is likely due to the benefits PA provides for mood, memory, focus and classroom behavior [14]. Increased levels of moderate-to-intense physical activity in childhood and adolescence are advantageous for both physical and mental well-being, which increases the possibility that AP will improve [15,16,17]. The majority of research shows that high school students who lead more active lifestyles [15,18,19,20,21] or who are more physically active [22,23] perform more effectively in school than students who lead more sedentary lifestyles. A study [24] demonstrated a direct correlation between engaging in PA and benefits, not just for physical health but also for AP. The authors emphasized that consistent participation in PA during educational years can influence lifelong habits in adulthood [24]. According to a study [25] involving high-school students from Finland, adolescents with high PA levels and reasonable amounts of time spent in sedentary behaviors were twice as likely to excel in school compared to their sedentary counterparts. In Portugal, students who engaged in higher levels of PA demonstrated a tendency to achieve better AP. Students with lower levels of PA exhibited reduced outcomes in their AP [26]. According to a Pelotas study that examined the connections between PA and academic and cognitive performance, adolescents who engaged in moderate levels of activity tended to achieve higher AP [27]. In a study conducted in Spain, the results showed a linear relationship between academic performance and physical activity; however, the connection between these two factors became even clearer when the data were analyzed in a different way, using a curved model instead of a straight line [15]. Moreover, there is a study that displayed a positive influence of vigorous PA and AP [28], and studies [25,29,30] that demonstrated improved AP as a result of increased PA levels. Additionally, recent systematic reviews have concluded that increased levels of PA and sports participation had a positive effect on AP in adolescents [31,32]. On the other hand, numerous studies do not show a positive influence of different PA levels on AP [33,34,35,36]. Due to the existence of mixed evidence in the aforementioned studies, it is important to conduct a regression analysis with the aim of delving into understanding how different levels of PA may influence AP.

Some studies have not found notable differences in the AP of physically active high-school adolescents compared to their less active peers [37,38,39], concluding that increased physical activity might not exert beneficial effects on AP. Notably, no studies have reported negative effects. While evidence suggests that physical activity can benefit AP, some studies have failed to support this theory [40,41,42], raising questions about the strength of this relationship [43]. Importantly, none of the studies cited in this review incorporated participants from southern Europe, particularly the Balkans. Thus, the findings of this study contribute to understanding this relationship in the Balkans, specifically, Serbia.

Exploring the connection between PA and AP in adolescents is important for various reasons. Firstly, adolescence is a formative period where habits and behaviors are established that can influence lifelong health and well-being. Understanding how PA impacts academic outcomes can inform policies and practices in schools to encourage a comprehensive approach to education that incorporates physical well-being. By identifying the benefits of PA on AP, educators and parents can design interventions that encourage active lifestyles, potentially leading to improved academic outcomes and overall student well-being. Furthermore, this knowledge can help address disparities in educational achievement by providing insights into how different levels of PA might affect student populations, leading to more equitable educational opportunities. To expand these discussions, the aim of this study was to ascertain if there is a relationship among PA levels and AP in adolescents from Serbia.

## 2. Materials and Methods

### 2.1. Study Design and Participants

This research used a stratified cluster sampling strategy and was a cross-sectional survey. We first sought out high school adolescents from one city and two municipalities. Conveniently, five high schools were chosen. Fourth grade students (average 17.9 ± 0.7 years old) were chosen from each school to participate in the research. The following requirements were set as inclusion criteria: the student had to consistently enroll in high school, provide a consent form that had been signed by both the student and their parents or guardians and complete the research instruments. The data from the questionaries were collected anonymously. Of the 950 pupils enrolled, 836 (88%) met these criteria, which is the final number of participants in the study. G*power software (latest ver. 3.1.9.7) [44] was used to calculate the sample size and statistical power, showing that with this number of participants we reached a 91.46% significance level.

### 2.2. Procedures

Following the request for authorization from the school administration to conduct the study on-site, the research participants were informed about the study’s objectives, data collection process, and were asked to take the consent form home for their parents/guardians to sign. The study adhered to the ethical guidelines of the 1964 Helsinki Declaration and complied with Resolution 466/12 of the Ministry of Health. Approval for the study was granted by the Research Ethics Committee of the Faculty of Sport and Physical Education in Niš, under protocol number 04-2035/2.

### 2.3. Physical Activity Levels

The short version of the International Physical Activity Questionnaire (IPAQ), validated for adolescent [45] use with an internal consistency of 0.64, was employed to collect data on time spent in physical activity. The IPAQ records all physical activities from the previous week. The data were then converted into metabolic equivalent of task (MET) units according to IPAQ guidelines: Walking MET-minutes/week = 3.3 × walking minutes/day × number of walking days/week; Moderate MET-minutes/week = 4.0 × moderate-intensity minutes/day × number of moderate-intensity days/week; Vigorous MET-minutes/week = 8.0 × vigorous-intensity minutes/day × number of vigorous-intensity days/week. Total physical activity MET-minutes/week was calculated by summing the walking, moderate, and vigorous MET-minutes/week scores.

### 2.4. Academic Performance

The Academic Performance Scale (APS), created by Carson Birchmeier, Emily Grattan, Sarah Hornbacher, and Christopher McGregory from Saginaw Valley State University, with a reliability coefficient of 0.89, was employed to evaluate students’ perceptions of their academic performance. The APS consisted of eight items on a 5-point Likert scale, asking participants to reflect on their behaviors as students. The scoring was categorized as follows: 33–40 for excellent performance; 25–32 for good performance; 17–24 for moderate performance; 9–16 for poor performance; and 0–8 for failing performance.

### 2.5. Statistical Analysis

All gathered data were analyzed using the Statistical Package for the Social Sciences, version 21.0 (IBM SPSS 21.0, SPSS Inc, Chicago, IL, USA). The Kolmogorov–Smirnov test (Table 1) indicated that the data did not follow a normal distribution. Frequencies of academic performance scale is shown in Table 2. Spearman’s correlation analysis (Table 3) is used to assess the relationship between physical activity levels and academic performance. The correlation coefficients were categorized as follows: weak (0 < r < 0.1), small (0.1 < r < 0.3), moderate (0.3 < r < 0.5), high (0.5 < r < 0.7), very high (0.7 < r < 0.9), and nearly perfect (0.9 < r < 1). Additionally, hierarchical regression analysis was conducted to evaluate the effect of physical activity levels on academic performance in adolescents, with vigorous intensity PA level in block 1, moderate intensity PA level in block 2, and light intensity PA level in block 3 (Table 4). A statistical significance level of *p* ≤ 0.05 was established.

## 3. Results

Table 1 presents the descriptive statistics of the physical activity levels and academic performance, along with the normality of the data distribution.

The data from Table 1 show that, on average, participants engaged in 1295.7 ± 1504.4 METs of vigorous intensity physical activity, 660.5 ± 894.5 METs of moderate intensity physical activity, and 1549.2 ± 1455.4 METs of light intensity physical activity. The total physical activity level across all intensities was 3504.4 ± 2736.4 METs. In terms of AP, participants scored an average of 25.9 ± 6.6 on the AP scale, which categorizes their performance as “good”. It is important to note that these results did not adhere to a normal distribution, as confirmed by the Kolmogorov–Smirnov test.

Based on the guidelines [46], students were divided into five categories according to their academic performance: excellent, good, moderate, poor and failing performance, as displayed in Table 2.

From 836 students, 168 (20.1%) had excellent performance, of which 333 (39.8%) had good performance. Moderate performance was achieved by 263 (31.5%) students, and 65 (7.8%) had poor performance. Only 7 (0.8%) of the students had failing performance.

The correlation between physical activity levels and academic performance is presented in Table 3.

The data indicated a significant correlation between vigorous, moderate, light physical activity and overall METs with AP, with *p*-values of 0.000, 0.005, 0.021 and 0.004, respectively. However, the correlation coefficients for these relationships were weak (0.127 for vigorous, 0.098 for moderate, 0.080 for light activity, and 0.181 for overall METs), meaning that while a statistical relationship exists, the strength of the association is very weak. In practical terms, this suggests that although adolescents who were more physically active tended to have better AP, the effect of physical activity on academic outcomes is minimal and may not be substantial in this sample. This finding highlights that while the association is statistically significant, its real-world relevance may be limited.

Hierarchical regression analysis, presented in Table 4, was conducted with variables organized into blocks according to their similarities. The findings indicate that all three models are statistically significant (*p* ≤ 0.05).

A hierarchical linear regression analysis for AP revealed that vigorous intensity physical activity in block 1 was significant (*R*^2^ = 0.101; *p* = 0.003), explaining 10.1% of the variance in AP. In block 2, both moderate and vigorous intensity physical activity significantly influenced AP (*R*^2^ = 0.183; *p* = 0.000), accounting for 18.3% of the variance. By comparing the results of block 1 and block 2, we can infer that moderate intensity physical activity contributed to 8.2% of the explained variance. Block 3 included vigorous, moderate and light intensity physical activity levels. By isolating the effects of vigorous and moderate intensity activity, we find that light intensity physical activity accounted for an additional 11.4% of the explained variance (*R*^2^ = 0.297; *p* = 0.000).

## 4. Discussion

This study sought to examine the relationship between physical activity (PA) and academic performance (AP) in adolescents from Serbia and additionally to ascertain whether and how much different levels of PA influence AP. The main findings suggested that PA showed a significant correlation with AP. Regarding the influence of PA levels on the AP of adolescents, it can be seen that the vigorous, moderate and light levels of PA significantly influence the AP.

In terms of the association between PA levels and AP, a noteworthy correlation was observed in the current investigation. As per the results from a Pelotas research study examining the connections between PA and academic as well as cognitive performance, adolescents engaging in various levels of PA demonstrate a tendency towards higher AP [27]. Another study [24] revealed a clear link between involvement in PA and its advantages, impacting not only physical well-being but also AP. It was underscored that regular engagement in PA throughout one’s adolescence could shape long-term behaviors in adulthood [24]. Based on the outcomes of our investigation, it is reasonable to infer that increased levels of PA can exert an influence on enhanced AP.

The results in the present study showed that moderate PA level and vigorous PA level are associated with AP, which are in accordance with a previous study [29]. A study involving English school students revealed that engaging in more hours of moderate-to-vigorous PA was linked with better AP [29]. Also, a study conducted in Portugal showed a trend of obtaining higher AP with those students who had higher PA level [26]. These circumstances underscore the imperative of enhancing PA within the school environment. It is notable that health organizations are actively advocating for the augmentation of PA during school hours.

The findings from our study revealed a correlation between light PA and AP. A significant portion of adolescents predominantly engage in light PA [47], yet there is a lack of studies investigating its effect on AP. Nonetheless, our results offer valuable insights into this relationship, contributing to a deeper comprehension of these variables. It is crucial to underscore the importance of establishing a connection between light PA and AP, particularly because of the sedentarism problem among adolescents [4].

However, certain studies have failed to identify substantial variances in the AP of active high school adolescents compared to those who are less active [37,38,39]. Also, while there is evidence that suggests PA is beneficial for PA, some studies have not found results that support these theories [40,41,42]. These research studies suggest that increased time devoted to PA might not necessarily yield positive impacts on AP, which differs from the results in our study The variations in outcomes may depend on the curriculum and programs of different high schools from different countries, considering that the curriculum of a high school depends on its type (general high school, vocational school, medical school).

The findings of this study revealed that vigorous intensity PA influenced AP by 10.1%. Furthermore, moderate intensity PA influenced AP by 8.2%, while light intensity PA influenced AP by 11.4%. Some studies are in accordance with the findings of our study [25,29,30] showing that a higher level of PA leads to higher AP. These results may stem from the assumption that PA can positively influence AP due to the improved organization and discipline that adolescents gain through various types of PA, demonstrating a positive transfer to later learning.

On the other hand, there are some studies that are not in line with our study [33,34,35], implying that there is no significant influence of PA levels and AP. The assumption is that greater duration of physical activity results in a lack of free time that could be used for studying [37,38,39], or that intense physical activity may lead to fatigue and subsequently to a lack of motivation for engaging in other activities.

In our study, we observed that regular PA does not appear to detrimentally affect AP; instead, it exhibits numerous positive impacts on the health of adolescents. For example, physical education classes serve as a catalyst for these benefits and present an opportune moment to instill an awareness of the positive impacts of PA and sports on health. Given that high-school students typically allocate a significant portion of their day to school, educational institutions represent an ideal environment to cultivate this awareness and foster understanding among high school students that engagement in sports can significantly contribute to their academic success.

The generalizability of the results may be constrained by the limited scope of the study sample, which primarily focused on a specific grade level within high schools. Future research could enhance the robustness of the study by expanding the sample to include participants from all high school grades, thereby providing a more thorough insight into the connection between PA and AP across different stages of adolescence. Moreover, another limitation is the lack of differentiation in results based on gender since the questionnaires did not include a question about sex. Examining the relationship between physical activity and academic performance by gender could reveal important patterns and enhance the relevance of future studies. Additionally, the study utilized self-report questionnaires to assess PA levels and AP; therefore, incorporating objective measures, such as PA testing and third-party evaluation of AP, could offer more accurate insights into participants’ activity levels. Furthermore, the current study adopted a cross-sectional design, which restricts the capacity to determine causal relationships between physical activity and academic performance. Conducting a longitudinal study would allow for the examination of changes in both PA levels and AP over time, offering a more nuanced understanding of their interplay.

## 5. Conclusions

The main findings in our study show that there is a relationship between physical activity levels and academic performance, reflecting a significant correlation and influence. Our study demonstrated that both moderate and vigorous levels of physical activity are linked with academic performance. Moreover, our findings suggest a connection between light physical activity and academic performance, although research on its influence on academic performance is limited. Considering that adolescents spend a substantial portion of their day sitting at school, educational institutions serve as opportune platforms to promote understanding among high school students that participation in sports or recreational activities can notably enhance their academic performance. Our findings suggest that integrating physical activity into adolescents’ daily routines can improve both their academic performance and overall health. Encouraging regular participation in moderate-to-vigorous activities, as well as promoting lighter activities and reducing sedentary behavior, can help foster long-term, healthy habits in students.

## Figures and Tables

**Table 1:** Descriptive statistics and data normality of distribution.

Variables	Mean ± Std. Dev.	K-S (Sig.)
Vigorous PA	1295.7 ± 1504.4	0.000
Moderate PA	660.5 ± 894.5	0.000
Light PA	1549.2 ± 1455.4	0.000
Overall METs	3504.4 ± 2736.4	0.000
APS	25.9 ± 6.6	0.001

Legend: K-S (Sig.)—Kolmogorov–Smirnov test; PA—physical activity; METs—metabolic equivalent of task, APS—Academic Performance Scale.

**Table 2:** Frequency table of academic performance scale.

Category	Frequency	Percent (%)
EP	168	20.1
GP	333	39.8
MP	263	31.5
PP	65	7.8
FP	7	0.8

Legend: EP—excellent performance; GP—good performance, MP—Moderate performance; PP—poor performance; FP—failing performance.

**Table 3:** Correlation between physical activity levels and academic performance.

		Vigorous PA	Moderate PA	Light PA	Overall MeTS
APS	*p*-value	0.000 **	0.005 *	0.021 *	0.004 *
	*r*-value	*r* 0.127	*r* 0.098	*r* 0.080	*r* 0.181
	CI (95%)	0.060, 0.193	0.030, 0.165	0.012, 0.147	0.115, 0.246

Legend: PA—physical activity; METs—metabolic equivalent of task, APS—Academic Performance Scale; *p* < 0.01 **; *p* < 0.05 *, *r*—correlation coefficient, CI (95%)—confidence interval upper and lower limit.

**Table 4:** Hierarchical regression analysis.

APS	F	ΔR^2^	R^2^	*p*
Block 1	8.595	0.099	0.101	0.003
Block 2	7.703	0.164	0.183	0.000
Block 3	8.155	0.258	0.297	0.000

Legend: F—F statistic; R^2^—coefficient of determination (square); ΔR^2^—adjusted R^2^; *p*—statistical significance; APS—Academic Performance Scale; Block 1—vigorous intensity physical activity level; Block 2—moderate intensity physical activity level, Block 3—light intensity physical activity level.

## Data Availability

The raw data supporting the conclusions of this article will be made available by the authors on request.
